# WT1 mRNA表达量对骨髓增生异常综合征诊断及预后评估的价值

**DOI:** 10.3760/cma.j.cn121090-20240507-00174

**Published:** 2024-10

**Authors:** 冰 李, 平 李, 瞄 苗, 苏宁 陈, 志坚 肖

**Affiliations:** 1 中国医学科学院血液病医院（中国医学科学院血液学研究所），血液与健康全国重点实验室，国家血液系统疾病临床医学研究中心，细胞生态海河实验室，天津 300020 State Key Laboratory of Experimental Hematology, National Clinical Research Center for Blood Diseases, Haihe Laboratory of Cell Ecosystem, Institute of Hematology & Blood Diseases Hospital, Chinese Academy of Medical Sciences & Peking Union Medical College, Tianjin 300020, China; 2 天津医学健康研究院，天津 301600 Tianjin Institutes of Health Science, Tianjin 301600, China; 3 大冢制药研发（北京）有限公司，北京 100005 Otsuka Beijing Research Institute, Beijing 100005, China; 4 苏州大学附属第一医院血液科，苏州 215006 Department of Hematology, The First Affiliated Hospital of Soochow University, Suzhou 215006, China

**Keywords:** 骨髓增生异常综合征, RNA，信使, 预后, 骨髓, 外周血, Myelodysplastic syndromes, RNA, messenger, Prognosis, Bone marrow, Peripheral blood

## Abstract

**目的:**

分析WT1 mRNA表达量对骨髓增生异常综合征（MDS）诊断及预后评估的价值。

**方法:**

此项多中心、前瞻性研究纳入国内8个临床试验中心403例MDS、疑似MDS和继发于MDS的急性髓系白血病（AML-MDS）患者，同时取外周血（PB）和骨髓（BM）样本，提取核酸，用WT1 mRNA测定试剂盒测定WT1 mRNA表达量。

**结果:**

PB和BM中WT1 mRNA表达量有很好的相关性（*r*＝0.778）。PB和BM中的WT1 mRNA表达量均随FAB或WHO（2008）分型的严重程度及IPSS-R或WPSS-R预后评分的增高而升高。MDS与AML-MDS患者PB和BM中WT1 mRNA的表达量差异有统计学意义（PB：3.11±0.98对4.57±0.53，*P*<0.05；BM：3.73±0.93对4.92±0.81，*P*<0.05）。IPSS-R相对低危组（极低危+低危）与相对高危组（中危+高危+极高危）MDS患者PB和BM中WT1 mRNA表达量的差异均有统计学意义（PB：2.60±0.76对3.48±0.91，*P*<0.05；BM：3.50±0.82对3.89±0.97，*P*<0.05）。IPSS-R相对低危组（极低危+低危+中危）与相对高危组（高危+极高危）MDS患者PB和BM中WT1 mRNA表达量的差异均有统计学意义（PB：2.82±0.89对3.61±0.85，*P*<0.05；BM：3.61±0.84对3.92±1.05，*P*<0.05）。WPSS-R相对低危组（极低危+低危+中危）和相对高危组（高危+极高危）MDS患者PB和BM中WT1 mRNA表达量的差异均有统计学意义（PB：2.56±0.79对3.61±0.82，*P*<0.05；BM：3.45±0.83对3.93±1.00，*P*<0.05）。

**结论:**

MDS患者PB与BM标本中WT1 mRNA的表达量有较好的相关性，且WT1 mRNA表达水平与MDS的疾病危险程度相关。

## Abstract (en)

**Objective:**

To analyze the value of the WT1 mRNA expression level in the diagnosis and prognostic evaluation of myelodysplastic syndrome（MDS）.

**Methods:**

A total of 403 patients with MDS, suspected MDS, and acute myeloid leukemia secondary to MDS（AML-MDS）from eight clinical trial centers in China were included in this multicenter, prospective study. Nucleic acid was extracted from the peripheral blood（PB）and bone marrow（BM）samples and WT1 mRNA expression was measured using the WT1 mRNA assay kit.

**Results:**

A good correlation（*r*＝0.778）was observed between the expression levels of WT1 mRNA in PB and BM. The expression levels of WT1 mRNA in both PB and BM increased with increasing FAB（French-American-British）or WHO（2008）（world health organization）classification scores, and increasing IPSS-R or WPSS-R prognostic scores. A statistically significant difference was observed in the expression levels of WT1 mRNA in PB and BM between MDS and AML-MDS patients（PB: 3.11±0.98 *vs* 4.57±0.53, *P*<0.05; BM: 3.73±0.93 *vs* 4.92±0.81, *P*<0.05）. A statistically significant difference also existed in the expression levels of WT1 mRNA in PB and BM between the IPSS-R relatively low-risk group（extremely low-risk + low-risk）and the relatively high-risk group（medium risk + high risk + extremely high risk）MDS patients（PB: 2.60±0.76 *vs* 3.48±0.91, *P*<0.05; BM: 3.50±0.82 *vs* 3.89±0.97, *P*<0.05）. Statistically significant differences were observed in the WT1 mRNA expression levels between the IPSS-R low-risk group（extremely low-risk + low-risk + moderate risk）and the high-risk group（high-risk + extremely high-risk）MDS patients in PB and BM（PB: 2.82±0.89 *vs* 3.61±0.85, *P*<0.05; BM: 3.61±0.84 *vs* 3.92±1.05, *P*<0.05）. Statistically significant differences were also observed in the expression levels of WT1 mRNA in PB and BM of MDS patients between the WPSS-R relatively low-risk group（extremely low-risk + low-risk + moderate risk）and the relatively high-risk group（high-risk + extremely high-risk）（PB: 2.56±0.79 *vs* 3.61±0.82, *P*<0.05; BM: 3.45±0.83 *vs* 3.93±1.00, *P*<0.05）.

**Conclusion:**

A good correlation was observed between the expression levels of WT1 mRNA in PB and BM specimens of MDS patients, and the expression level of WT1 mRNA is related to the disease risk of MDS.

既往研究表明，威尔姆斯肿瘤基因1（WT1）的表达水平可作为骨髓增生异常综合征（MDS）患者预后、疾病进展监测的重要指标，是缺乏细胞遗传学和分子生物学资料的MDS患者病情严重程度和预后分组的辅助指标[1]–[5]。WT1 mRNA试剂盒已于2013年5月在日本获批上市，用于MDS的辅助诊断及病程进展监测[6]–[9]，现将该试剂盒在中国上市的临床试验结果报道如下，重点分析MDS患者外周血（PB）和骨髓（BM）中WT1 mRNA表达量的相关性，以及WT1 mRNA表达量与诊断分型及预后的相关性。

## 病例与方法

1. 病例：本临床试验纳入2014年6月至2015年7月全国8个临床试验中心（中国医学科学院血液病医院、首都医科大学附属北京同仁医院、第二军医大学附属长海医院、苏州大学附属第一医院、南方医科大学南方医院、天津医科大学总医院、中南大学湘雅三医院、中南大学湘雅医院）的患者，获得了中国医学科学院血液病医院伦理委员会批准（批件号：SJ2014003-EC-1），征得了患者本人知情同意并签署知情同意书。主要入选标准：（1）符合以下任何1项诊断标准的患者：①已确诊的MDS患者；②疑似MDS患者；③继发于MDS的急性髓系白血病（AML-MDS）患者。（2）年龄18～70周岁，性别不限。主要排除标准：接受过造血干细胞移植的患者；合并其他活动性肿瘤的患者；既往骨髓检查中发生干抽现象的患者；研究者认为不适合的患者；孕妇、哺乳期妇女或可能妊娠的患者。

2. 诊断：将外周血涂片、骨髓穿刺涂片、骨髓穿刺涂片铁染色及骨髓活检切片HE染色标本集中交给MDS审评委员会专家进行审评，根据FAB分型和WHO（2008）分型进行分型诊断[10]–[11]。研究者依据审评专家诊断的分型结果，应用国际预后积分系统修订版（IPSS-R）和基于WHO分类的预后评分系统修订版（WPSS-R）对患者进行预后评分[12]–[13]。

3. WT1 mRNA检测：采集PB（7.0 ml）和BM（1.0 ml）样本，48 h内送至中心实验室，采用QIAamp RNA试剂盒（Cat：52304）（德国QIAGEN公司产品）提取mRNA。使用WT1 mRNA检测试剂盒（日本大冢公司产品）对WT1 mRNA进行定量检测。计算患者WT1 mRNA表达量（拷贝/µg RNA），进行对数转换并计算平均值±标准差，再将对数转换值还原成常数。

4. 统计学处理：MDS各疾病类型组间WT1 mRNA表达量的比较采用WT1 mRNA表达量（拷贝/µg RNA）的对数转换值，并进行Tukey-Kramer HSD检验，*P*<0.05为差异有统计学意义。相关性分析采用皮尔森相关系数。

## 结果

1. 一般资料：研究共纳入403例患者，其中2例患者提前退出。401例患者按照FAB标准分型，MDS患者177例，分为4个亚型：难治性贫血（RA，103例）、难治性贫血伴有环状铁粒幼红细胞（RARS，8例）、难治性贫血伴有原始细胞过多（RAEB，59例）、转化中RAEB（RAEB-t，7例）；按照WHO（2008）分型，MDS患者178例，分为7个亚型：难治性血细胞减少伴单系发育异常/难治性贫血/难治性中性粒细胞减少/难治性血小板减少（RCUD/RA/RN/RT，20例）、RARS（4例）、难治性血细胞减少伴多系发育异常（RCMD，88例）、难治性贫血伴原始细胞增多-1（RAEB-1，30例）、难治性贫血伴原始细胞增多-2（RAEB-2，32例）、MDS-未分类（MDS-U，3例）、MDS伴单纯5q−（1例）。根据FAB和WHO（2008）分型标准，AML-MDS患者14例［其中6例完全缓解（CR）］，急性髓系白血病（AML）患者17例（其中3例CR），再生障碍性贫血（AA）患者14例。

2. PB和BM中WT1 mRNA表达量的相关性：390例患者PB和BM中WT1 mRNA表达量的回归直线为*y*＝0.721*x*+1.423，皮尔森相关系数*r*＝0.778，*P*<0.001（图1A）。从390例患者中剔除提取RNA时调整后浓度<5 ng/µl的99例患者，剩余291例患者PB和BM中WT1 mRNA表达量的回归直线为*y*＝0.715*x*+1.473，皮尔森相关系数*r*＝0.806，*P*<0.001（图1B）。

**图1:**
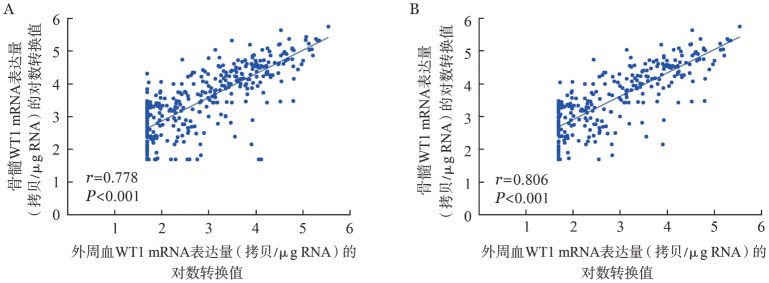
390例（A）和291例（B）患者外周血和骨髓WT1 mRNA表达量的相关性

3. PB和BM中WT1 mRNA表达量（表1）：按FAB标准进行分型诊断，MDS与AML-MDS患者PB、BM标本的WT1 mRNA对数表达量的差异均有统计学意义（PB：3.11±0.98对4.57±0.53，*P*<0.05；BM：3.73±0.93对4.92±0.81，*P*<0.05）。

**表1:** MDS、AML-MDS、AA患者外周血和骨髓标本WT1 mRNA的对数表达量（*x*±*s*）

疾病类型	外周血		骨髓	
	例数	WT1 mRNA对数表达量	例数	WT1 mRNA对数表达量
MDS	175	3.11±0.98	175	3.73±0.93
AML-MDS	8	4.57±0.53	8	4.92±0.81
AML-MDS（达到CR）	6	4.46±0.55	6	4.56±0.77
AML	12	4.16±0.94	14	4.32±1.08
AML（达到CR）	3	3.43±0.32	3	3.49±0.07
AA	14	1.86±0.41	14	2.68±0.50

**注** MDS：骨髓增生异常综合征；AML-MDS：继发于MDS的急性髓系白血病；AA：再生障碍性贫血；CR：完全缓解；AML：急性髓系白血病

4. MDS各亚型的WT1 mRNA表达量：根据FAB分型，RA、RARS、RAEB、RAEB-t、AML-MDS患者PB和BM中WT1 mRNA表达量呈升高趋势（图2A、B）。在PB标本中，RA和RAEB、RA和RAEB-t、RA和AML-MDS、RARS和RAEB-t、RARS和AML-MDS、RAEB和AML-MDS间WT1 mRNA表达量的差异均有统计学意义（*P*值均<0.05）；在BM标本中，RA和RAEB、RA和RAEB-t、RA和AML-MDS、RARS和RAEB、RARS和RAEB-t、RARS和AML-MDS、RAEB-t和AML-MDS间WT1 mRNA表达量的差异均有统计学意义（*P*值均<0.05）。

**图2:**
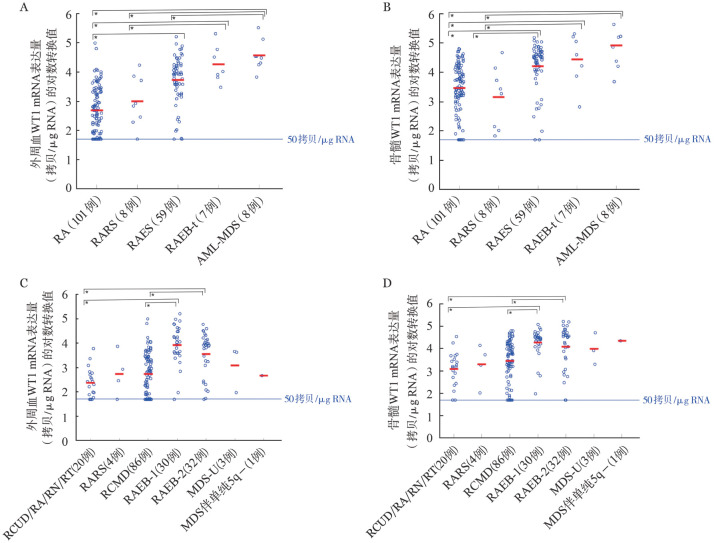
骨髓增生异常综合征各亚型患者外周血（PB）和骨髓（BM）中WT1 mRNA表达量 **A** FAB分型，PB标本；**B** FAB分型，BM标本；**C** WHO（2008）分型，PB标本；**D** WHO（2008）分型，BM标本；*Tukey-Kramer HSD检验显示*P*<0.05；RA：难治性贫血；RARS：难治性贫血伴有环状铁粒幼红细胞；RAEB：难治性贫血伴有原始细胞过多；RAEB-t：转化中RAEB；RCUD：难治性血细胞减少伴单系发育异常；RN：难治性中性粒细胞减少；RT：难治性血小板减少；RCMD：难治性血细胞减少伴多系发育异常；RAEB-1：难治性贫血伴原始细胞增多-1；RAEB-2：难治性贫血伴原始细胞增多-2；MDS-U：MDS-未分类

根据WHO（2008）分型，MDS各组患者PB和BM中WT1 mRNA表达量见表2。PB和BM中WT1 mRNA表达量均呈现出随严重程度增高而升高的趋势（图2C和2D）。PB和BM标本中RCUD/RA/RN/RT和RAEB-1、RCUD/RA/RN/RT和RAEB-2、RCMD和RAEB-1、RCMD和RAEB-2间WT1 mRNA表达量的差异均有统计学差异（*P*值均<0.05）。

**表2:** 不同预后评分系统各风险组骨髓增生异常综合征患者的WT1 mRNA对数表达量（*x*±*s*）

预后评分系统	各风险组（年龄校正后）	外周血			骨髓		
		例数	WT1 mRNA对数表达量	*P*值	例数	WT1 mRNA对数表达量	*P*值
IPSS-R	相对低危组（极低危+低危）	69	2.60±0.76	<0.05	69	3.50±0.82	<0.05
	相对高危组（中危+高危+极高危）	91	3.48±0.91		91	3.89±0.97	
IPSS-R	相对低危组（极低危+低危+中危）	104	2.82±0.89	<0.05	104	3.61±0.84	<0.05
	相对高危组（高危+极高危）	56	3.61±0.85		56	3.92±1.05	
WPSS-R	相对低危组（极低危+低危+中危）	87	2.56±0.79	<0.05	88	3.45±0.83	<0.05
	相对高危组（高危+极高危）	75	3.61±0.82		74	3.93±1.00	

**注** IPSS-R：国际预后积分系统修订版；WPSS-R：WHO分型预后积分系统修订版

5. 不同预后评分风险组WT1 mRNA表达量：依据IPSS-R预后评分标准，MDS患者分为极低危、低危、中危、高危和极高危组。PB和BM中WT1 mRNA表达量呈现出随着IPSS-R危险级别增高而升高的趋势。IPSS-R预后评分极低危和中危、极低危和高危、极低危和极高危、低危和中危、低危和高危、低危和极高危患者PB中WT1 mRNA表达量的差异均有统计学意义（*P*值均<0.05）。IPSS-R相对低危组（极低危+低危）和相对高危组（中危+高危+极高危）患者PB和BM的WT1 mRNA表达量差异均有统计学意义（PB：2.60±0.76对3.48±0.91，*P*<0.05；BM：3.50±0.82对3.89±0.97，*P*<0.05）。IPSS-R相对低危组（极低危+低危+中危）和相对高危组（高危+极高危）患者PB和BM中WT1 mRNA表达量的差异均有统计学意义（PB：2.82±0.89对3.61±0.85，*P*<0.05；BM：3.61±0.84对3.92±1.05，*P*<0.05）。

依据WPSS-R预后评分标准，MDS患者分为极低危、低危、中危、高危和极高危组。PB和BM中WT1 mRNA的表达量呈现出随着WPSS-R预后危险分层的增高而升高的趋势。WPSS-R预后评分中极低危和高危、极低危和极高危、低危和高危、低危和极高危、中危和高危、中危和极高危患者PB中WT1 mRNA表达量的差异均有统计学意义（*P*值均<0.05），极低危和高危、极低危和极高危、低危和高危组患者BM中WT1 mRNA的表达量的差异均有统计学意义（*P*值均<0.05）。WPSS-R相对低危组（极低危+低危+中危）和相对高危组（高危+极高危）患者PB和BM中WT1 mRNA表达量的差异均有统计学意义（PB：2.56±0.79对3.61±0.82，*P*<0.05；BM：3.45±0.83对3.93±1.00，*P*<0.05）（表2）。

6. 染色体核型与WT1 mRNA表达量的相关性：162例MDS患者按照IPSS-R预后评分中的染色体核型分为极好、好、中、差、极差组，结果显示，随着染色体核型预后变差，PB和BM中WT1 mRNA表达量呈增高趋势（表3）；163例MDS患者按照WPSS-R预后评分中的染色体核型分为好、中、差组，结果显示，好、中、差组PB中WT1 mRNA表达量逐渐增高（表4），核型“好”和核型“差”组的差异有统计学意义（2.87±1.00对3.39±0.82，*P*<0.05）。

**表3:** IPSS-R染色体核型不同分组患者的WT1 mRNA对数表达量（*x*±*s*）

染色体核型	外周血		骨髓	
	例数	WT1 mRNA对数表达量	例数	WT1 mRNA对数表达量
极好	2	2.53±1.19	2	3.43±1.29
好	98	2.92±0.98	98	3.68±0.90
中	30	3.41±0.79	30	3.97±0.74
差	16	3.29±0.90	16	3.72±1.07
极差	16	3.41±0.83	16	3.50±1.14

**注** IPSS-R：国际预后积分系统修订版

**表4:** WPSS-R染色体核型不同分组患者的WT1 mRNA对数表达量（*x*±*s*）

染色体核型	外周血		骨髓	
	例数	WT1 mRNA对数表达量	例数	WT1 mRNA对数表达量
好	99	2.87±1.00	99	3.60±0.94
中	35	3.24±0.82	35	3.88±0.79
差	29	3.39±0.82	29	3.64±1.09

**注** WPSS-R：WHO分型预后积分系统修订版；染色体核型“好”组和“差”组比较，*P*<0.05

7. WT1 mRNA表达量对RA和AA的鉴别诊断：根据14例AA患者与101例RA患者的WT1 mRNA表达量绘制患者特征曲线（ROC），确定鉴别诊断的临界值，分析结果显示，PB中WT1 mRNA临界值设定为150拷贝/µg较为合理，此时的检测灵敏度为67％（68/101），特异度为93％（13/14）（图3A）；BM中WT1 mRNA的临界值设定为1 200拷贝/µg较为合理，此时的灵敏度为75％（76/101），特异度为86％（12/14）（图3B）。

**图3:**
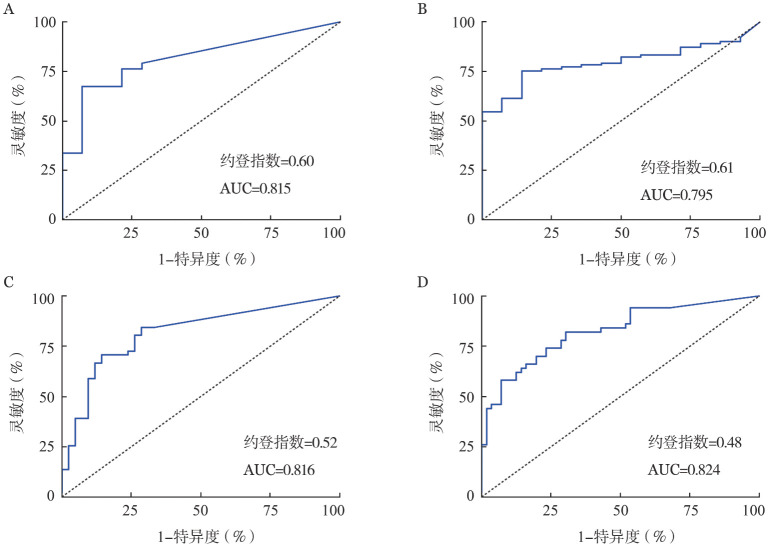
难治性贫血（RA）和再生障碍性贫血（AA）患者外周血（PB）和骨髓（BM）中WT1 mRNA表达量的ROC分析 **A** AA（14例）与RA（101例）患者PB中WT1 mRNA表达量的ROC曲线；**B** AA（14例）与RA（101例）患者BM中WT1 mRNA表达量的ROC曲线；**C** AA（56例）与RA（50例）患者PB中WT1 mRNA表达量的ROC曲线；**D** AA（56例）与RA（50例）患者BM中WT1 mRNA表达量的ROC曲线

鉴于上述AA患者的例数只有14例，追加了一项对56例AA患者和50例RA患者WT1 mRNA表达量的分析，AA与RA患者PB和BM中WT1 mRNA表达量的差异均有统计学意义（PB：1.80±0.59对2.67±0.83，*P*<0.001；BM：2.29±0.75对3.30±0.80，*P*<0.001）。ROC分析结果显示，PB中WT1 mRNA临界值设定为150拷贝/µg较为合理，此时的检测灵敏度为71％（36/51），特异度为81％（34/42）（图3C）；BM中WT1 mRNA的临界值设定为1 200拷贝/µg时的检测灵敏度为66％（32/49），特异度为82％（46/56）（图3D）。

## 讨论

MDS表现为无效造血、难治性血细胞减少，高风险向AML转化。诊疗时评估其向AML转化的风险，并根据结果确定治疗方案尤为重要。在目前发现的参与MDS发病的突变基因中，WT1 mRNA表达量可以反映MDS的疾病进展阶段[1]–[6]，并与IPSS评分有良好的相关性。WT1 mRNA检测可作为预测MDS病情发展、判断疗效、指导临床治疗的指标。

目前我国医疗单位或实验室进行WT1检测时大多采集BM，检测结果主要以WT1与本中心ABL等内参基因绝对拷贝数比值的相对表达水平为主，检测结果不便于在不同实验室间进行比较，且检测步骤较多，历时较长，限制了WT1检测技术在我国MDS诊断及常规监测中的广泛应用。本研究所用检测试剂盒应用实时定量PCR原理，采用“一步法”快速定量PB或BM中的WT1 mRNA，用于MDS或AML的辅助诊断以及病情进展的监测[6]–[9]。

Tamura等[3]将69例MDS患者分为3组进行回顾性分析，结果显示，PB中WT1 mRNA高表达组的生存时间和无病生存时间均较低表达组缩短，PB中WT1 mRNA的表达水平是MDS的独立预后因素。Du等[5]报道，PB中WT1 mRNA表达水平反映接受去甲基化药物治疗的MDS患者的疾病进展。应用本试剂盒检测MDS患者PB中WT1 mRNA的表达量是阿扎胞苷治疗MDS疗效的预测因素[6]，可用于评估MDS患者的预后[7]。

本研究结果证实本试剂盒适用于PB和BM两种样本，且患者PB和BM中WT1 mRNA表达量有较好的相关性，提示PB可取代BM进行WT1 mRNA检测。与BM采样相比，PB采样操作简单、侵入性小，Kitamura等[8]建议采用对患者创伤较小的PB样本监测WT1 mRNA表达水平，在诊断或怀疑复发等情况下，使用形态学异常的BM样本进行检测。

本研究中患者PB和BM的WT1 mRNA表达量随MDS疾病严重程度的增加而升高，随IPSS-R、WPSS-R预后评分危险度的增加而升高，随染色体核型预后变差而升高，可作为MDS预后评估的辅助标志物，与既往研究结果一致[7]。

RA是MDS疾病早期阶段的一个亚型，其临床表现、实验室检查、骨髓细胞形态学与AA有相似之处，在临床诊断中容易混淆[14]。虽有研究提示骨髓小粒、集落培养、细胞因子、骨髓涂片和骨髓病理等检查有助于二者的鉴别诊断，但均为单中心、小样本量、回顾性形态学或非定量研究结论[15]–[17]。本研究结果显示，PB中WT1 mRNA表达量的临界值设定为150拷贝/µg，BM中设定为1 200拷贝/µg时，有助于鉴别RA和AA，为WT1 mRNA检测用于RA和AA的鉴别诊断提供了证据。已完成的临床研究显示，638名国内健康人中594名PB中WT1 mRNA表达量不到50拷贝/µg，阴性率为93.10％。

本研究验证了中国成人MDS或疑似MDS患者PB或BM中WT1 mRNA表达量与MDS不同亚型、预后评分、病情进展的相关性，可辅助MDS诊断及监测其病情进展，有助于患者个体化治疗方案的制订。
